# Inhibition of HIV-1 Viral Infection by an Engineered CRISPR Csy4 RNA Endoribonuclease

**DOI:** 10.1371/journal.pone.0141335

**Published:** 2015-10-23

**Authors:** Rui Guo, Hong Wang, Jiuwei Cui, Guanjun Wang, Wei Li, Ji-Fan Hu

**Affiliations:** 1 Stem Cell and Cancer Center, First Hospital, Jilin University, Changchun, China; 2 Stanford University Medical School, Palo Alto, California, 94304, United States of America; George Mason University, UNITED STATES

## Abstract

The bacterial defense system CRISPR (clustered regularly interspaced short palindromic repeats) has been explored as a powerful tool to edit genomic elements. In this study, we test the potential of CRISPR Csy4 RNA endoribonuclease for targeting HIV-1. We fused human codon-optimized Csy4 endoribonuclease with VPR, a HIV-1 viral preintegration complex protein. An HIV-1 cell model was modified to allow quantitative detection of active virus production. We found that the *trans*-expressing VPR-Csy4 almost completely blocked viral infection in two target cell lines (SupT1, Ghost). In the MAGI cell assay, where the HIV-1 LTR β-galactosidase is expressed under the control of the *tat* gene from an integrated provirus, VPR-Csy4 significantly blocked the activity of the provirus-activated HIV-1 reporter. This proof-of-concept study demonstrates that Csy4 endoribonuclease is a promising tool that could be tailored further to target HIV-1.

## Introduction

In spite of recent advances in therapy, HIV-1, the causative agent of AIDS, continues to be a major public health threat as the global pandemic continues to spread, particularly in the developing world. There are 30 million adults and children living with HIV, and there are 1.8 million new HIV infections each year throughout the world[1]. Current therapy remains very expensive, and more importantly, it cannot completely cure the disease[2–4], highlighting the urgency of pursuing new strategies to find a cure to control HIV infection.

Intracellular gene therapy has been explored as a promising approach to control HIV infection (see reviews[5–8]), including siRNA, intrabodies, HIV entry targeting, endonucleases, as well as tailored Cre recombinase [9,10]. Cre recombinase is a tyrosine recombinase enzyme derived from the P1 bacteriophage. The enzyme uses a topoisomerase I-like mechanism to carry out site-specific recombination in the target DNA. The 38kDa recombinase recognizes a 34-bp double-stranded hairpin DNA sequence known as loxP and catalyses the recombination event between two loxP sites. The loxP site consists of two 13 bp palindromic sequences flanking an 8bp spacer region. Buchholz’s group[11] used a substrate-linked protein evolution approach to successfully engineer Cre recombinase to recombine a sequence present in the LTRs of an integrated provirus. The evolved recombinase Tre, when expressed in primary CD4+ T cells, excises integrated HIV proviral DNA from the genome of infected cells[11–14] and suppresses viral replication[15].

In prokaryotes, the clustered regularly interspaced short palindromic repeats (CRISPRs) defense system confers resistance to invasive genetic elements. The bacterial immune system synthesizes CRISPR-derived RNAs (crRNAs) from the fragments of foreign DNAs that are integrated into the CRISPR loci[16], serving as homing oligonucleotides to guide CRISPR-associated (Cas) protein enzymes to degrade invading viruses harboring cognate sequences[17,18]. Among them, the CRISPR/Cas9 system has been recently explored as a powerful tool in genome editing with high specificity and low cell toxicity[19,20].

CRISPR Csy4 is a RNA endoribonuclease that processes CRISPR transcripts (pre-crRNAs) in Pseudomonas aeruginosa[21]. Csy4 binds to its cognate RNA in the major groove of the crRNA repeat and cleaves pre-crRNAs using serine and histidine residues in the active site[22–24]. Considering the success of the Cre recombinase, we became interested in exploring the potential of the Csy4 RNA endoribonuclease. Csy4 is a site-specific RNA endoribonuclease that recognizes a cognate hairpin sequence that is as short as 18 bp (Cy18)[24]. We hypothesized that it may be possible to engineer Csy4 to destroy HIV-1 RNA. Tailoring Csy4 to recognize the sequence present in the 5′-LTR and 3′-LTR of HIV-1 would represent a novel approach to target HIV-1. We thus became interested in exploring the potential of the Csy4 defense system to serve as a therapeutic tool in targeting the HIV-1 LTR. In this proof-of-concept study, we examined the potential of Csy4 ribonuclease in inhibiting RNA viral infection using two HIV-1 reporter systems[25,26].

## Materials and Methods

### Cell lines and plasmids

The following three cell lines were obtained through the NIH AIDS Reagent Program, Division of AIDS, NIAID, NIH. SupT1 cell line expresses high levels of surface CD4 and is useful in studies of cytopathic effects of HIV-1[27]. P4R5-MAGI cell line stably expresses human CCR-5, CD4, and β-galactosidase under the control of HIV-1 LTR, which can be transactivated by HIV TAT. Infection with HIV can be detected by β-gal staining[26]. Ghost(R3/X4/R5) cell line can be used to titer virus and evaluate drug sensitivities[28]. Two HIV-1 vectors (RGH-WT[25] and pLAI.2[29]) were obtained as a courtesy of the MRC AIDS Directed Program.

The viral packaging 293T cell line was purchased from American Type Culture Collection (ATCC)(Manassas, VA).

### HIV vector construction

To construct the HIV-Cy28 vector, a 28bp containing the Csy4 recognizing RNA hairpin was amplified by primers JH2408 (forward): 5-CGAGCTGTACAAGTAGGCTCGAGTTCACTGCCGTATAGGCAGCTA-3’ and JH2409 (reverse): 5’-TCCATGTTTTTCCAGGTCTTTCTTAGCTGCCTATACGGCAGTGAAC-3’. The PCR product was cloned into RGH-WT vector at the Xho1 restriction site with Choo-Choo Cloning Kits (McLab, CA). For the HIV-Ct28, we replaced the 28bp Csy4 hairpin with a 28bp random oligonucleotide. The plasmid sequences were confirmed by DNA sequencing.

To construct the VPR-Csy4 vector, Csy4 sequencing was first human codon-optimized and synthesized by IDT Integrated DNA Technologies (Coralville, IO). The HIV-1 VPR and PC cleavage domains were amplified, respectively, from pLAI.2 using forward primer JH2301: 5’- TCGACGGTACCGCGGGCCCgccaccATGgaacaagccccagaag-3’ and reverse primer JH2085: 5’-TAGTACTTTCCTGATTCCAGCACTGACCAACCCATCTACTTGTTCggatctactggctccatttcttgc-3’. Cys4 was amplified by forward primer JH2410: 5-CAGTGCTGGAATCAGGAAAGTACTAGACCATTACCTGGATATCCGACTG-3’ and reverse primer JH2411: 5-TGATCTAGAGTCGCGGCCGCTTAAAACCAAGGGACGAATCCCCCCTTGCTCAG 3’. Both the VPR and Csy4 fragments were ligated by overlapping PCR and cloned into the Apa1 and Not1 sites in EGFP-N1 vector (Clontech, CA).

For the Csy4 construct, the codon-optimized Csy4 was amplified with PCR and joined with the CMV promoter at the 5’-end and with the TK-poly A signal at the 3’-end. The expression cassette was inserted into a modified pGreenPuro vector (SBI, CA) to generate lentiviral vector Cys4. We then used PCR to introduce a H19A mutation in Csy4 to construct the mutated version of Csy4 (mCsy4).

For the Csy4-Puro and mCsy4-Puro constructs, the Csy4 or mCsy4 cassette was amplified and inserted into pCDH1 (SBI, CA) to generate the Csy4-Puro or mCsy4-Puro vector.

The Cy28 control vector was constructed by inserting the Csy4 28bp hairpin into GreenPuro vector between BamH1 and EcoR1 restriction sites to generate Cy28-pGreenPuro (pCy28). For Csy4-Cy28 and Csy4-Ct28 vector, the Cys4 28bp hairpin and a random 28bp sequence were amplified and inserted into Csy4-GreenPuro at the BamH1 and EcoR1 restriction sites, respectively.

### Plasmid construction and viral packaging

**Fig 1:**
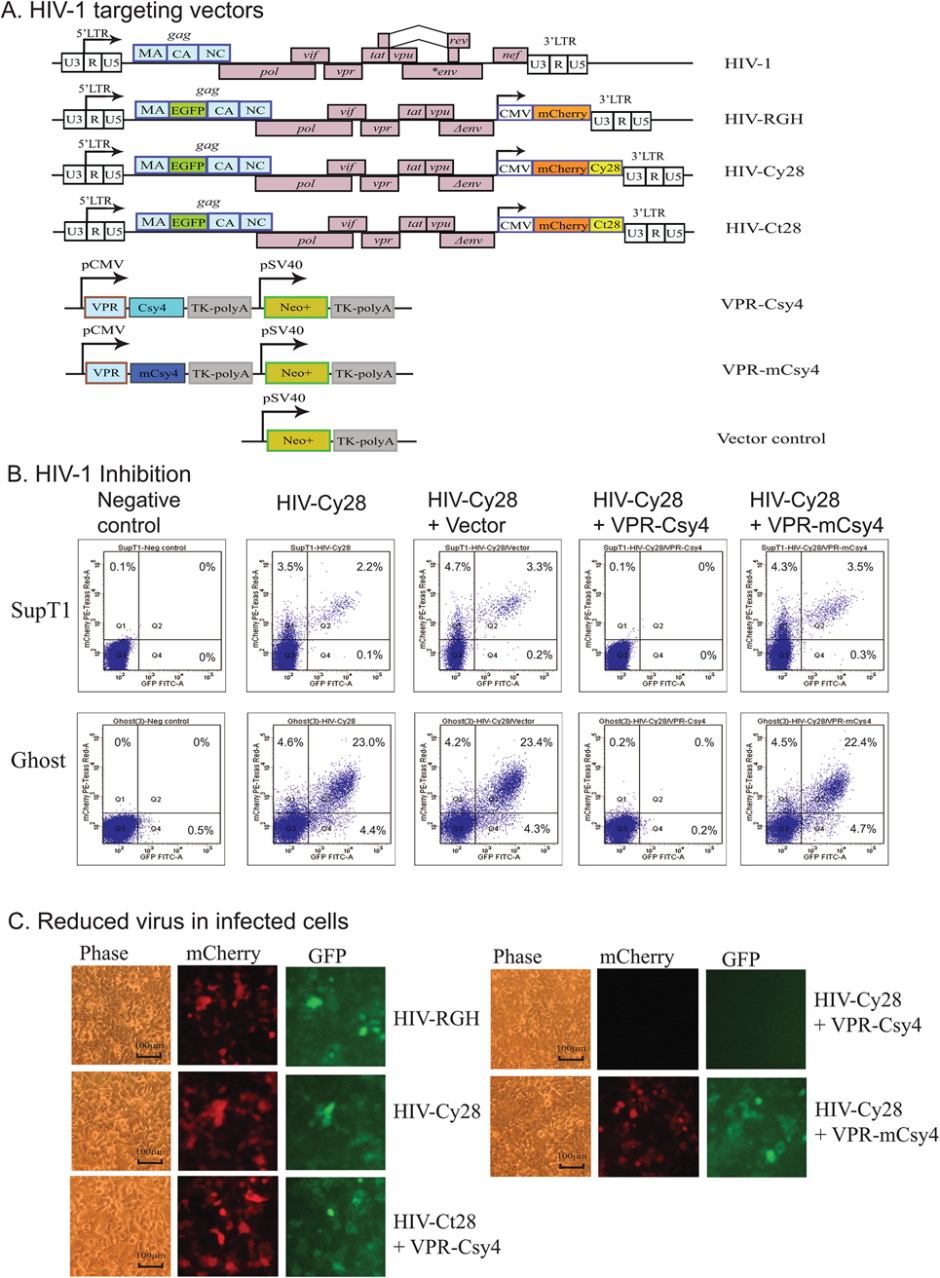
Inhibition of HIV-1 infection by Csy4 endoribonuclease. **A.** HIV-1 Csy4 targeting vectors. HIV-1: HIV-1 gene structure; HIV-RGH: the HIV-1 reporter vector; HIV-Cy28: the HIV-RGH vector containing a 28bp Csy4-binding hairpin downstream of the mCherry reporter gene; HIV-Ct28: the HIV-RGH vector containing a random 28bp sequence; VPR-Csy4: the VPR-Csy4 fusion protein; VPR-mCsy4: the vector containing the mutated Csy4; Vector control: a control vector that does not contain the Csy4 endoribonuclease. The HIV-1 VPR domain drives the packaging of Csy4 into the HIV-1, where the endoribonuclease binds to and degrades the HIV-1 RNA virus, leading to the blockage of the viral infection. B. Inhibition of HIV-1 infection by Csy4 in SupT1 and Ghost cells. HIV-positive cells were analyzed by FACS 72 hours after HIV-1 infection. mCherry-positive/EGFP (LTR)-negative cells: latently infected cells; EGFP-positive cells: actively infected cells; mCherry/EGFP double-positive cells: active HIV-1 infection with the integrated provirus. Note the significantly reduced infection of HIV-1 in the VPR-Csy4 treatment group in all three infected cells. Negative control: uninfected cells. C. Reduction of integrated HIV-1 provirus in infected 293T cells. HIV-1 virions were detected by EGFP fluorescence and integrated viruses were detected by mCherry fluorescence.

Two HIV-1 vectors (RGH-WT, pLAI.2), obtained from NIH AIDS Reagent Program, were used as the backbone to construct our vectors (**Fig 1A**). To generate HIV-1 pseudovirus, modified RGH plasmids were co-transfected with VSV-G packaging plasmid into 293T cells with Lipofectamine 2000 (Invitrogen, CA) following the protocol provided by the manufacturer. For lentivirus packaging, constructed plasmids were co-transfected with pSPAX2 and pMD2.G packing vectors using Lipofectamine 2000. The virus supernatants were collected at 48h and 72h after transfection [30].

### HIV-1 p24 Antigen Capture Assay by ELISA

HIV pseudo-virus in the supernatant was tittered by HIV-1 p24 Antigen Capture Assay (ABLinc, Cat. #5421) following the manufacturer’s manual. Briefly, the viral supernatant was diluted and run in parallel with HIV-1 p24 standards. After washing, the plate was incubated with 100μl conjugate solution at 37°C for 60min, reacted with 100μl peroxidase substrate, and recorded at 450nm for the HIV-1 p24 antigen absorbance. After quantitation, equal titers of viruses were used for cell infection.

### Pseudo HIV virus infection assay

Target cells (SupT1 and GHOST) were seeded at appropriate cell density to assure that the cells reached 70%-90% confluence by the following morning. For GHOST cells, the supernatant containing equal titers of HIV-1 was mixed with 1μl polybrene (10 mg/ml) in 1.5ml DMEM media, and transferred them onto the target cells. For SupT1 suspension cells, we transferred 2x10^5^ cells into a 15 ml conical tube. After adding equal titers of HIV-1 containing 10 mg/ml polybrene, cells were centrifugation at 800g for 30 minutes at 32°C. Cell pellets were suspended in 2 ml fresh media and cultured in 6-well plates for three days. The HIV-1-infected cells expressed GFP and mCherry fluorescence, and infection was analyzed by flow cytometry (LSRFORTESSA, BD).

### Assessment of HIV-1 virion infection by the MAGI cell assay

Infectivity of HIV-1 virions was also assessed using the MAGI cell assay. In this assay, integration of viral DNA results in the eventual appearance of easily visible multinucleated blue syncytia after ß-Gal staining. After normalized by viral core protein p24 quantitation, equal titers of viruses were used infect P4R5-MAGI cells [26]. Seventy-two hours post HIV-1 viral infection, cells were washed three times with PBS and fixed with 4% formaldehyde for 5 min at room temperature. After rinsing twice with PBS, cells were stained with 5-bromo-4-chloro-3-indolyl-b-D-galactopyranoside (X-gal) staining solution in a humidified chamber at 37°C overnight. After the removal of the staining solution, cells were rinsed in PBS and photographs were taken under a microscope.

Viral infection in MAGI cells was also quantitated by the measurement of β-galactosidase activity using the Promega assay system (cat# E2000; Promega, CA). Forty-eight hours after the transfection, cells were lysed and incubated with 50μl 2X assay buffer at 37°C for 30 minutes. The absorbance of β-galactosidase was read at 410 nm in a plate reader.

### Quantification of virus-associated gene expression by real time qPCR

After removing the residual genomic and plasmid DNAs with DNase I (Sigma, MO), viral RNAs were converted into cDNA using M-MLV reverse transcriptase (Invitrogen, CA) as described [31,32]. For qPCR, cDNA samples were amplified by SYBR PrimeScript™ RT-PCR Kit using CFX96^TM^ real-time system (BIO-RAD, CA). Genes analyzed included virus-associated mRNAs (mCherry, *GAG*, *POL*, *VSVG*, *and REV*) and the IFN anti-virus pathway genes (*IFNα2*, *IFNα6*, *APOBEC3F*, *and APOBEC3G*). Expression was quantitated by normalizing against β-actin as the housekeeping gene[33,34]. The viral DNAs (mCherry, *GAG*, *POL*, *VSVG*, *and REV*) were quantitated by qPCR. PCR primers used for qPCR are listed in **S1 Table**.

### HIV-1 integration assay

A two-step transfection approach was used to examine if Csy4 works at the proviral integration level. Briefly, the wild type Csy4 and the mutated Csy4 (mCsy4) carrying the H19A mutation were cloned in lentiviral cloning vector, packaged in 293T cells, and used to transfect Ghost and 293T cells. After transfection, stable cell clones were selected using 1mg/ml puromycin and used for the second infection with HIV-1 viruses.

HIV-1 pseudoviruses were packaged in 293T cells using the HIV-RGH system in the absence of Csy4. Viral supernatants were collected and equal titers of HIV-1 pseudoviruses were used to transfect the Cys4- and mCys4-stable cells. Forty-eight hours after the transfection, the HIV-1 infected cells were quantified for GFP and mCherry using cytometry (BD Biosciences, CA).

### Statistical analysis

The statistical significance of differences between treatment groups was assessed using SPSS 21.0 software (SPSS, Inc., IL). One-way ANOVA (Bonferroni test) was used to compare variables among treatment groups. All experiments and assays were repeated 3 times and data were expressed as the mean±SEM of independent experiments. The values were considered significantly different at p < 0.05.

## Results

### The *trans* inhibition of HIV-1 viral infection by Csy4 RNA endoribonuclease

Csy4 ribonuclease recognizes its short cognate hairpin RNA sequence and specifically cuts the target RNA[24]. Using a hairpin homolog search using RNAfold webserver (http://rna.tbi.univie.ac.at/cgi-bin/RNAfold.cgi), we found that both the HIV-1 5’LTR and the 3’LTR contain a putative Csy4 targeting site (CTS, **S1A Fig**). This CTS sequence contains a unique stem and loop structure that is homologous to the Csy4 hairpin RNA (right panel). As substrate-linked protein evolution approaches have successfully been used to redirect Cre DNA recombinase to recognize the HIV-1 LTR DNA[11], we hypothesized that Csy4 ribonuclease could be also engineered to recognize the homologous CTS site in the HIV-1 LTR by using the same protein evolution approach (**S1B Fig**).

To evaluate the therapeutic potential of Csy4 RNA endoribonuclease, we adopted a novel double-labeled HIV-1 reporter system (HIV-RGH)[25] in this proof-of-concept study. This RGH reporter system allows the quantitative detection of infected cells containing both silent and productive HIV-1 pseudovirus (**Fig 1A**, HIV-RGH). In this reporter system, the EGFP reporter gene is inserted in the *gag* and is flanked by HIV-1 protease cleavage sites. Upon viral integration, the Gag-EGFP cassette is under the control of the HIV-1 promoter, thus serving as a quantitative marker for HIV-1 LTR gene activity. In addition, the vector also contains the mCherry reporter gene in the *nef* locus under the control of the CMV immediate-early promoter. As a result, mCherry is expressed constitutively in infected cells, allowing quantitative detection of the integrated virus.

Using this reporter system, virus-infected cells can easily be detected by FACS analysis. We modified this HIV-1 reporter system by inserting a 28bp Csy4 hairpin-containing sequence (Cy28) in front of the 3’-LTR (**Fig 1A**, HIV-1 Cy28). It has been reported that VPR, a HIV-1 virion-associated accessory protein, can guide foreign proteins to the HIV particle[35–37]. Therefore, we expressed Csy4 endoribonuclease as a VPR-Csy4 fusion protein. With this approach, the *trans*-expressing Csy4 RNA endoribonuclease was expressed and incorporated in the HIV virions, where it can degrade the viral RNA.

To observe the effect of Csy4, we produced viral stocks in 293T cells. Pseudoviruses in the supernatants were quantitated by p24 ELISA kit. After adjustment, equal titers of viruses were used to infect target cells (SupT1, Ghost). SupT1 is a non-Hodgkin's T cell lymphoma line that expresses high levels of surface CD4[38]. SupT1 cells are useful in studies of cell fusion and cytopathic effects of HIV-1. GHOST cells express HIV receptor and co-receptors CCR3, CXCR4, and CCR5 on the cell surface[28]. These cells can be used to evaluate drug sensitivities.

After viral infection, we used FACS to detect the HIV-1 infected cells. The HIV-Cy28 viral stock contained the active HIV-1. As expected, after infection, both EGFP- and mCherry-positive cells were detected in SupT1 and Ghost cells (**Fig 1B**, the HIV-Cy28 group). Two control treatments (HIV-Cy28/vector and HIV-Cy28/mCsy4) did not affect HIV-1 infectivity. In the treated group (HIV-Cy28/VPR-Csy4), however, we found that the *trans* expressing VPR-Csy4 dramatically inhibited the active HIV-1. The VPR-Csy4-mediated inhibition was so potent that essentially no active HIV-1were detected in treated cells as compared with that in the vector control cells (negative control group). Similarly, HIV-1 viral fluorescence was barely detected in VPR-Csy4 treated cells using a fluorescent microscope (**Fig 1C**).

### Csy4 inhibits HIV1 infection in the MAGI cell assay

The MAGI cell assay has been widely used to characterize the biological activity of HIV-1 integrase in viral infection. MAGI cells contain a β-galactosidase gene (β-gal) under the control of an HIV-1 LTR. Activation of the LTR β-gal cassette requires the viral *tat* transactivator from an integrated provirus. Thus, infection with HIV-1 can be detected simply by β-gal staining [39]. This reporter system is thus useful to monitor HIV-1 infection.

**Fig 2:**
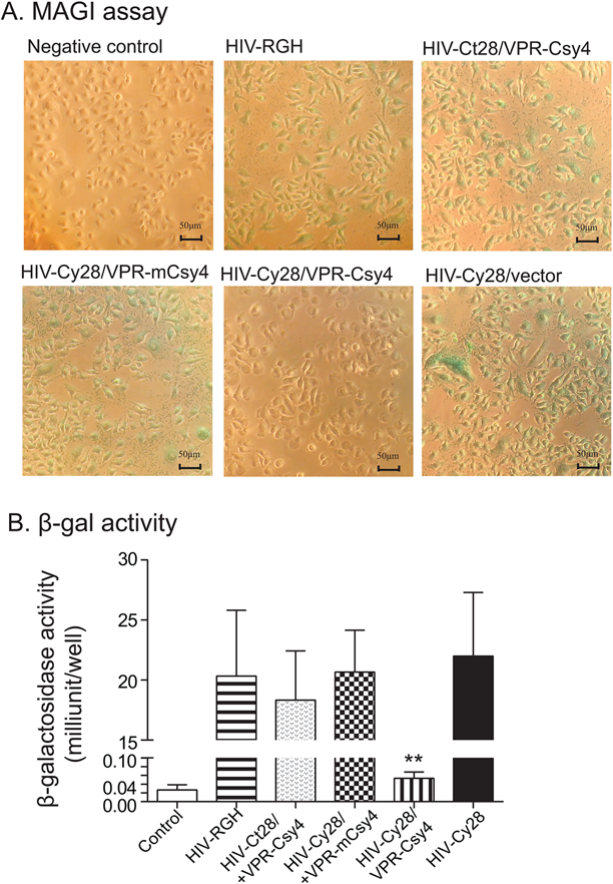
Csy4 inhibits HIV-1 infection in MAGI assays. The MAGI cells contain a stably integrated β-galactosidase gene (β-gal) under the control of an HIV-1 LTR. Integration of viral DNA results in activation of the LTR β-gal cassette. Seventy-two hours post HIV-1 viral infection, P4R5-MAGI cells were stained with X-gal. Infection of HIV-1 results in the formation of blue nuclei. A: MAGI assay staining. Negative control: Control MAGI cells that were not infected with HIV-1 pseudovirus; HIV-RGH: Cells infected with the HIV-RGH vector as the positive control; HIV-Ct28/VPR-Csy4: Cells infected with HIV-1 containing Ct28 random control and Csy4; HIV-Cy28/VPR-mCsy4: Cells infected with HIV-1 containing the Cy28 for Csy4 binding and the mutated mCsy4; HIV-Cy28/VPR-Csy4: Cells infected with HIV-1 containing the Cy28 for Csy4 binding and were treated by Csy4 (treatment group); HIV-Cy28/vector: Cells infected with HIV-1 containing the Cy28 for Csy4 binding but were not treated with Csy4 (vector control). B: Quantitation of virus-infected MAGI cells using β-Galactosidase enzyme assay. All data shown are mean±SEM from three independent experiments. ** p<0.01 as compared with controls.

We used this reporter system to confirm the role of Csy4 in inhibiting HIV-1 infectivity. The HIV-1 infected MAGI cells were stained blue for β-gal, which is activated by infection with HIV-1 virions. The HIV-Cy28 viral stock also generated β-gal positive MAGI cells throughout the field. However, β-gal positive cells were rarely detected in the Csy4-treated group. The mutated Csy4 did not affect viral infection. The HIV-Cy28 group had slightly but not statistically significantly higher β-gal activity than did the HIV-RGH group (**Fig 2A**). We also used a β-galactosidase kit to quantitate the infection of HIV-1 in MAGI cells. Similarly, we found that treatment with VPR-Csy4 significantly inhibited viral infection in MAGI cells (**Fig 2B**, p<0.01). These data suggest that the RNA endoribonuclease is able to block viral infection.

### Csy4 blocks lentiviral infection in *cis*

**Fig 3:**
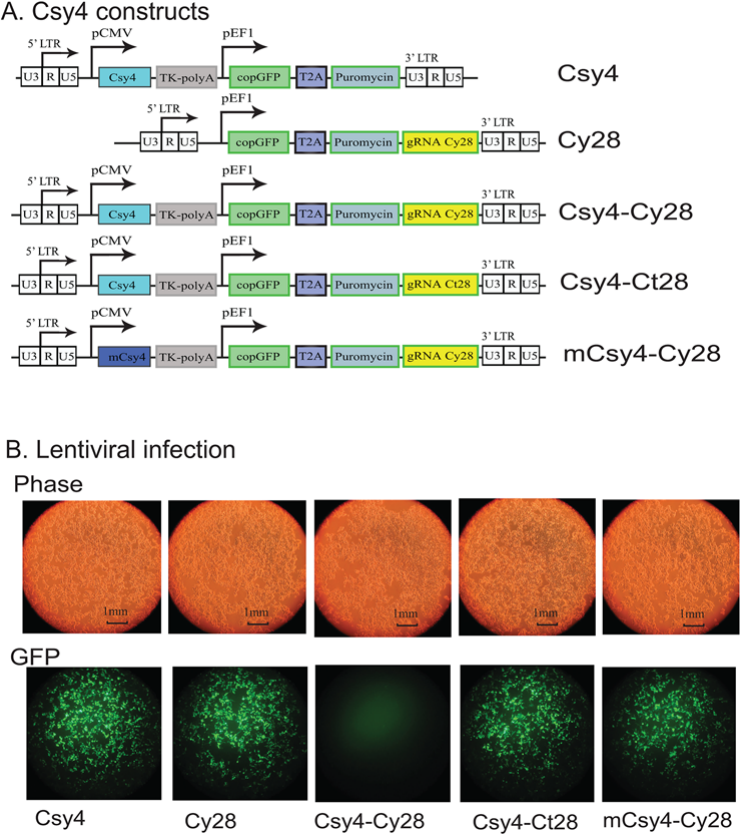
Csy4-mediated inhibition of lentiviral infection. A. Schematic diagram of Csy4 viral targeting vectors. Csy4: the Cys4 endoribonuclease control vector that lacks the Csy4 hairpin binding site; Cy28: the Cys4 endoribonuclease vector containing the 28 bp Csy4 hairpin binding site near the viral LTR; Csy4-Cy28: the targeting vector containing both the Cys4 endoribonuclease and the Csy4 hairpin binding site in the viral LTR (treatment group); Csy4-Ct28: control vectors containing the Cys4 and a random 28 bp control sequence in the viral LTR; mCsy4-Cy28: control vectors containing the mutated Csy4 endoribonuclease and a 28 bp sequence in the viral LTR. B. Inhibition of lentiviral infection by Csy4 in 293T cells. Viral infection was analyzed by EGFP florescence in 293T cells 48h after viral infection. Note the specific inhibition of lentiviral infection by Csy4 endoribonuclease in the Csy4-Cy28 treatment group, where Csy4 binds to the hairpin in the LTR and degrades the viral RNA.

To further delineate the antiviral role of Csy4, we expressed the endoribonuclease in *cis* in a lentiviral vector that contains the HIV-1 backbone. The viral transfer vector carried copGFP as the indicator for viral infection (**Fig 3A**, Csy4 group). The Csy4-deleted vector was used as the vector control (Csy28 group). The vectors containing Csy4 and a 28bp random oligonucleotide (Ct28) were used as Csy4 hairpin controls (Csy4-Ct28 group). The mCsy4-Cy28 treatment was used as an enzyme control.

Using this system, we observed active viral infection with copGFP fluorescence in all four control groups (**Fig 3B**, Csy4, Cy28, Csy4-Ct28, mCsy4-Cy28). In the treated group (Csy4-Cy28), however, we found that the cis-expressed Csy4 effectively inhibited viral infection in 293T cells. The inhibition by Csy4 was very potent and no viral fluorescence was detected in the treatment group. The mutated Csy4 (mCsy4) did not show significant inhibition of viral infection.

### Viral packaging machinery is interrupted by Csy4 endoribonuclease

**Fig 4:**
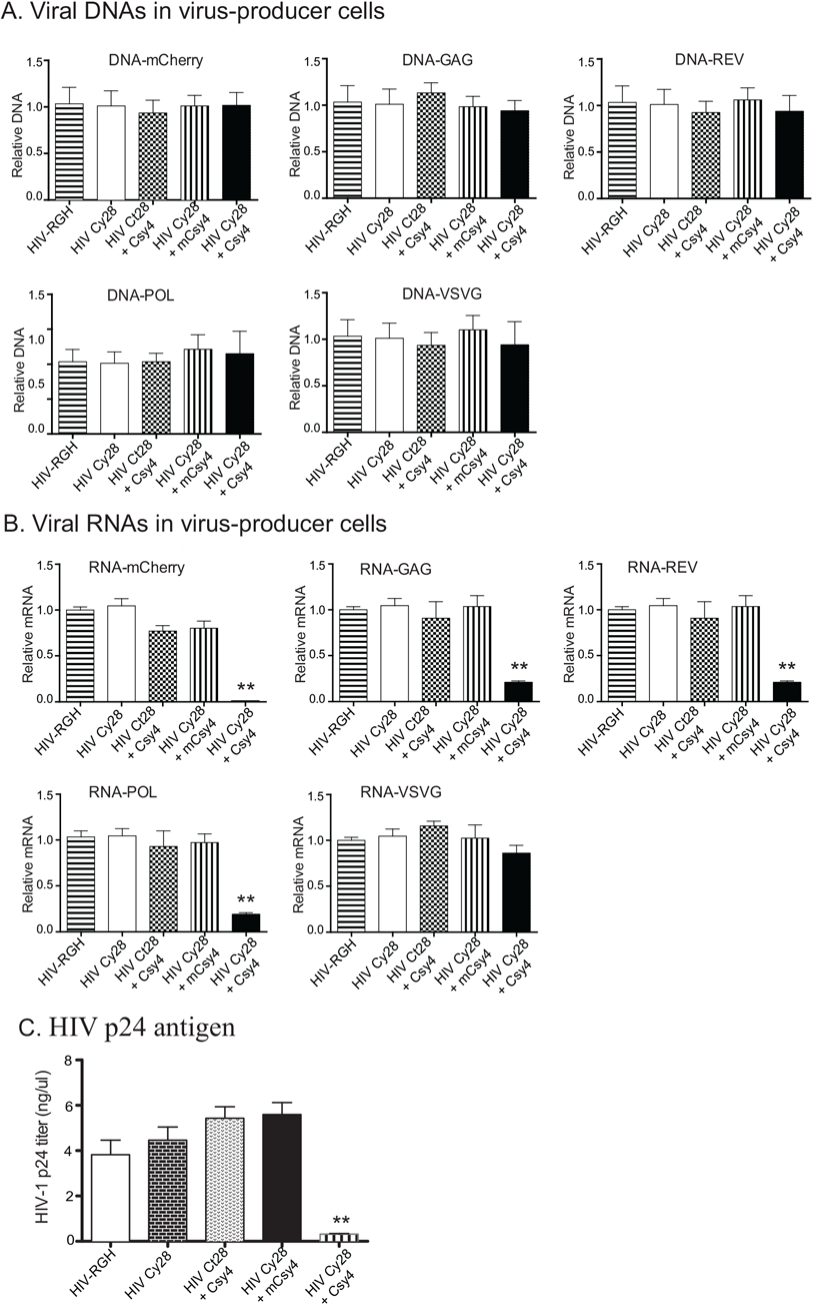
Expression analysis of the viral packaging components. A. Quantitation of the viral gene DNAs in 293T packaging cells. VSVG: the viral packaging gene that encodes of the vesicular stomatitis virus G glycoprotein; GAG: the HIV-1 gene that encodes the P24 non-glycosylated capsid protein CA; REV: the HIV-1 gene that encodes glycosylated transmembrane envelope protein; POL: the HIV-1 gene that encodes the RNA polymerase; mCherry: the vector control. All data shown are mean±SEM from three independent experiments by normalization over controls. B. Expression of viral package genes. Total RNA was extracted 48 hours after viral packaging in 293T cells and was used to analyze the expression of viral packaging genes. β-ACTIN was used the PCR control. ** p<0.01 as compared with normal controls. C. Production of HIV-1 by HIV-1 p24 antigen capture assay. The core of the HIV-1 virion is made up of two strands of RNA and various proteins including the p24 core antigen. The HIV-1 p24 antigen capture assay is a double-antibody sandwich enzyme immunoassay used to quantitate HIV-1 p24 in tissue culture samples. HIV-1 was quantitated 48h after viral packaging in 293T cells. ** p<0.01 as compared with normal controls.

We then used the HIV-RGH system to examine potential mechanisms underlying HIV inhibition by Csy4 RNA endoribonuclease. We first used real-time PCR to quantitate the viral packaging gene DNAs in 293T packaging cells. Csy4 is a RNA endoribonuclease. As expected, we did not detect any significant differences in viral packaging gene DNAs in the Cy28+Csy4 treatment group compared with the four control groups (**Fig 4A**).

We then used RT-qPCR to compare viral gene RNAs in packaging cells. We observed a reduction of viral RNAs (viral mCherry, GAG, POL, and REV) in Cy28+Csy4 treated cells as compared with the four control groups (**Fig 4B**, HIV-RGH, HIV-Cy28, HIV-Ct28+Csy4, HIV-Cy28+mCsy4). Viral RNAs were slightly but not significantly lower in the Ct28/Csy4 group than in the Cy28 group.

We then used a HIV-1 p24 antigen capture assay to titer the HIV-1 in the supernatants. The HIV-1 titers were 3.75 ng/μl, 4.60 ng/μl, 5.61 ng/μl, and 5.82 ng/μl in groups HIV-RGH, HIV–Cy28, HIV-Ct28+Csy4, and HIV-Cy28+mCsy4 respectively. However, the production of HIV-1 virions was reduced to 0.31 ng/μl in the Csy4-treated group (**Fig 4C**, p<0.01). Together, these data suggest that Csy4 dramatically inhibits HIV-1 viral packaging.

### Csy4 does not affect the host antiviral system

**Fig 5:**
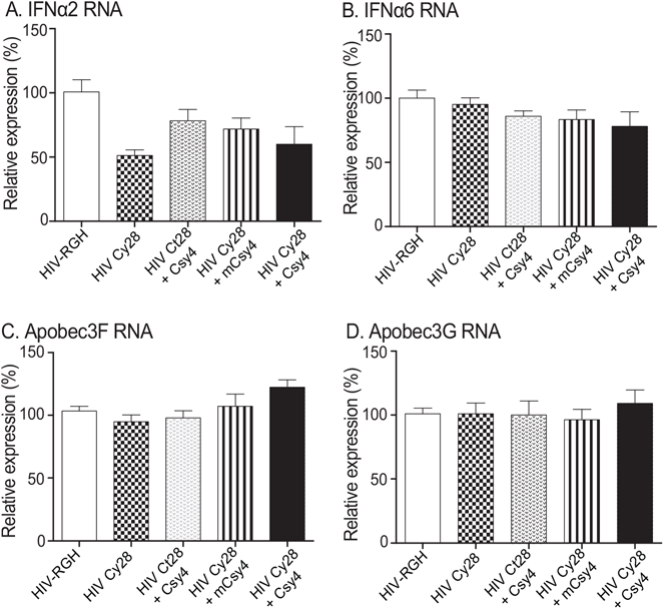
Expression of innate defense genes. Forty-eight hours after viral infection, Ghost cells were collected for RNA analysis using real-time PCR. All data shown are mean±SEM from three independent experiments by normalization over controls.

In order to learn if Csy4 affected viral infection through the host defense system, we used RT-qPCR to quantitate the expression of interferon α2, α6, and two host viral restriction factors (APOBEC3F and APOBEC3G). As seen in **Fig 5**, Csy4 does not affect the expression of these host factors involved in antiviral defense.

### Csy4 inhibits HIV-1 by preventing proviral DNA integration

**Fig 6:**
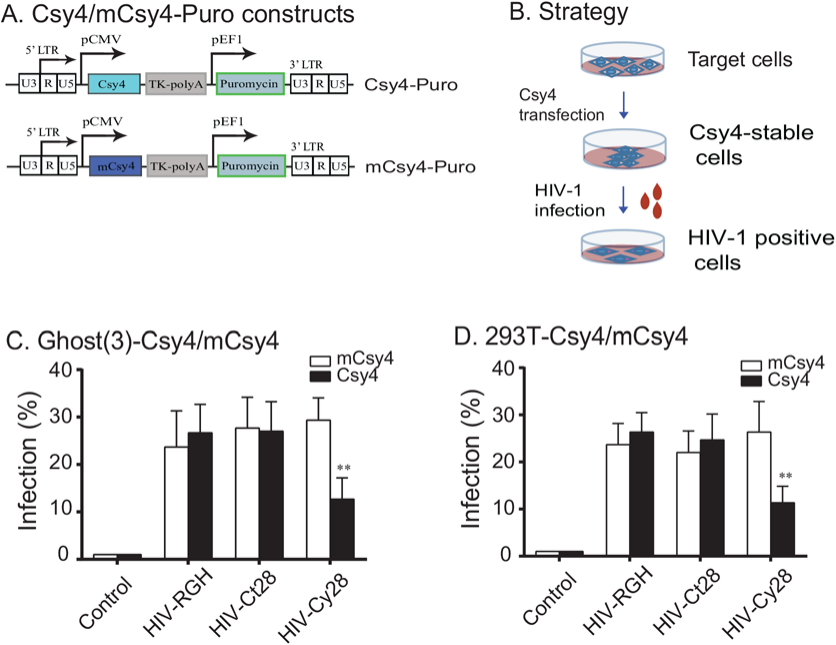
Inhibition of HIV-1 infection in Csy4-stable cells. A. Diagram of the Csy4 and mCsy4 vector. B. Strategy to examine the inhibition of Csy4 on the HIV-1 preintegration in the HIV-RGH system. Target cells were first transfected with the Csy4 lentivirus. Csy4 stable cell clones were selected and transfected by equal titers of HIV-1 viruses. The mCherry/EGFP double-positive cells were quantitated by FACS as the HIV-1 positive cells. C-D. Inhibition of HIV-1 provirus in Csy4-stable Ghost cells (C) and 293T (D) cells. All data shown are mean±SEM from three independent experiments by normalization over controls. ** p<0.01 as compared with the mutated Csy4 (mCsy4) group.

We then focused on the integration of HIV-1 provirus in infected cells. To avoid the effect of Csy4 on viral packaging, we employed a two-step strategy to infect target cells (**Fig 6A and 6B**). To avoid the degradation of HIV-1 RNA at viral packaging, Csy4 and HIV-1 viruses were packaged separately. Two target cell lines (Ghost and 293T) were first transfected with Csy4 or its mutated construct (mCsy4). After transfection, stable cell clones were selected by puromycin and were used for the second infection with equal titers of HIV-1 pseudoviruses.

Seventy-two hours after HIV-1 pseudovirus infection, cells were collected for FACS analysis of the HIV-1-positive cells. In both HIV-RGH and HIV-Ct28 groups, ~ 25% of the Ghost cells were positive for HIV-1 in the Csy4-stable and mCsy4-stable cells (**Fig 6C**). In the treatment group (HIV-Cy28), however, the stably-transfected Csy4 significantly reduced HIV-1 infection as compared with the mCsy4 (p<0.01). Inhibition of viral infection was also observed in 293T cells (**Fig 6D**). Mutated Csy4 did not affect HIV-1 infection. These data suggest that Csy4 also blocks viral infection at the level of retroviral infection and provirus integration.

## Discussion

Gene therapy for HIV-1 infection is a potentially rewarding approach for long-term control of this chronic disease. In this proof-of-concept study, we examined the potency of Csy4 in inhibiting HIV-1 infection. To target the HIV-1 RNA, we inserted a 28bp fragment that contains the Csy4 binding site (Cy18) into the immediate upstream of the 3′-LTR of HIV-1. We delivered Csy4 RNA endoribonuclease into the HIV-1 virions by linking it to VPR, a HIV-1 virion-associated accessory protein VPR that is able to guide foreign proteins to the HIV particle[35–37]. Using this approach, we demonstrate that Csy4 RNA endoribonuclease is very potent in inhibiting HIV-1 infection in SupT1 and Ghost cell lines. The activity of the fusion enzyme is so potent that neither the active nor the inactive HIV-1 provirus can be detected in treated cells. These data suggest that if the viral RNA molecule contains a single Cy18 sequence, Csy4 is able to eradicate it within the cell.

A number of intracellular immunization strategies have shown initial promise in both *in intro* and *in vivo* studies. These experiments employ a variety of mechanisms involving the use of transdominant proteins [1–3], decoys [3–7], ribozymes [5, 8–12], antisense constructs and siRNAs. siRNA-based constructs have received increased attention due to their target specificity and improved potency. Numerous laboratories have tested a variety of anti-HIV siRNAs designed to target both viral and cellular genes that are essential for viral infection. Csy4 RNA endoribonuclease can be tailored using a substrate-linked protein evolution approach that has been successfully used to tailor the Cre recombinase[11]. Using library screening, we hope to identify a Csy4 variant that specifically recognizes the hairpin homolog present in the HIV-1 5′-LTR and 3′-LTR sequence. As compared with existing approaches, this enzyme degrades its target directly at the RNA level. Thus, it is possible that a tailored Csy4 could serve as gene therapy against HIV infection. Future studies will be necessary to engineer Csy4 and redirect it to the putative target site in the HIV-1 LTR. It will also be important to examine its efficiency and duration in patients’ primary cells as reported by Dahabieh et al [25].

Existing highly active antiretroviral therapy (HAART) has diminished the morbidity of HIV-infected individuals However, current therapies only suppress the viral life cycle without eradicating the infection. In addition, new strains of HIV-1 are emerging that are resistant to suppressive treatments. The engineered Csy4, however, can be used as a promising *ex vivo* therapy in T cells or CD34 cells. When delivered into these target cells by lentivirus, the enzyme will consistently be expressed, thus offering an attractive approach to treat HIV-1 infection. Csy4 is a site-specific RNA endoribonuclease that recognizes a minimum 18bp hairpin (Cy18) in the target RNA. Accordingly, we did not observe any cytotoxic effect in the Csy4-expressing host cells, including cell growth and proliferation. Although directly participating in a prokaryote defense system that confers resistance to invasive genetic elements[21], Csy4 itself did not significantly alter cellular innate defense genes, including interferon alpha and viral restriction factors Apobec3. These preliminary data may pave the way for developing Csy4 as a potential anti-HIV tool.

It should be emphasized that this report constitutes a proof-of-concept study for Csy4 as a potential HIV-therapeutic. Many challenges need to be overcome before the Csy4 nuclease can be engineered to recognize the HIV-1 LTR. For example, can Csy4 be successfully engineered using the substrate-linked protein evolution approach [11]? Can the engineered Csy4 specifically target the HIV-1 LTR without causing “off target” effects? Can the HIV-1 LTR truly fold into an independent hairpin for targeting? What is the likelihood that other hairpins will also be targeted non-specifically by the enzyme? Will the engineered Csy4 be functional in T cells or CD34 cells? Most importantly, will the enzyme to be able to function without adverse side effects in a clinical setting? Future studies are needed to address these critical questions. Nevertheless, the results of this study offer an early proof of principle for this type of approach, thus forming a useful basis for the development of future HIV therapies.

In summary, this proof-of-concept study demonstrates that Csy4 RNA endoribonuclease may serve as a promising tool to target HIV-1. The *trans*-expressing Csy4 is able to destroy the HIV-1 RNA virus when it contains a single Csy4 binding site. Csy4 destroys HIV-1 RNA primarily at the level of provirus production and partially at the provirus preintegration. In Csy4-treated cells, neither HIV-1 pseudovirus nor provirus can be detected. We are in the process of tailoring Csy4 to specifically recognize the HIV-1 LTR hairpin sequence, hoping that some day this therapy could be developed as a novel therapeutic reagent in *ex vivo* cell studies to protect against HIV infection.

## Supporting Information

S1 Fig HIV-1 targeting by Csy4 endoribonuclease. Putative HIV-1 targeting sequence of Csy4 endoribonuclease. The putative Csy4 targeting site in HIV-1 LTR was predicted by RNAfold webserver (http://rna.tbi.univie.ac.at/cgi-bin/RNAfold.cgi). Csy4 ribonuclease specifically cuts the target RNA at its short cognate hairpin RNA sequence. The RNAfold homolog search revealed that the HIV-1 LTR contains a unique stem and loop structure (CTS) that is homologous to the Csy4 hairpin RNA, suggesting that it could serve as a putative targeting site for Csy4 endoribonuclease. LTR: long terminal repeat sequence (U3-R-U5). Both the 5’LTR and the 3’LTR of HIV-1 contain a putative Csy4 targeting site (CTS), consisting of a stem (green) and a loop (red). The CTS RNA is homologous to the Csy4 hairpin RNA. Red arrow: Csy4 cutting site (**A**). Degradation of HIV-1 by Csy4 endoribonuclease. After binding to the putative targeting sequences, Csy4 degrades the HIV-1 5’- and 3’-LTR, leading to the degradation of the virus either at the viral packaging or proviral integration (**B**).
(EPS)

S1 Table PCR primers used for viral packaging gene DNA and RNA. (DOCX)

S2 Table PCR primers used for the expression of the IFN pathway genes. (DOCX)
