# Supplementary material for: Inhibition of HIV-1 Viral Infection by an Engineered CRISPR Csy4 RNA Endoribonuclease
Source: PLoS One. 2015 Oct 23;10(10):e0141335. doi: 10.1371/journal.pone.0141335 (PMC4619743; doi:10.1371/journal.pone.0141335)
Supplement: S1 Table — (DOCX) [file pone.0141335.s002.docx]

**S1 Table. PCR primers used for viral packaging gene DNA and RNA**

| Lenti (WPRE) | JH135 | Tctttatgaggagttgtggcccgttg |
| --- | --- | --- |
|  | JH136 | aaagtcccggaaaggagctgac |
| GAG | JH1619 | taagcgggggagaattagatcg |
|  | JH1620 | CCAGGATTAACTGCGAATCGTT |
| REV | JH1621 | CCATTAGGAGTAGCACCCACCA |
|  | JH1622 | CTGCGCCCATAGTGCTTCCT |
| POL | JH2045 | cagtactggatgtgggcgatgc |
|  | JH2046 | ccctgtggaagcacattgtactg |
| VSVG | JH2048 | tggggagtcagactcccatcag |
|  | JH2049 | gtcctgaattagacttacatccactg |
