# Supplementary material for: Inhibition of HIV-1 Viral Infection by an Engineered CRISPR Csy4 RNA Endoribonuclease
Source: PLoS One. 2015 Oct 23;10(10):e0141335. doi: 10.1371/journal.pone.0141335 (PMC4619743; doi:10.1371/journal.pone.0141335)
Supplement: S2 Table — (DOCX) [file pone.0141335.s003.docx]

**S2 Table. PCR primers used for the expression of the IFN pathway genes**

| IFN a2 | JH2060 | cagcctgggtagcagga |
| --- | --- | --- |
|  | JH2061 | ttcagatttccccaggaggag |
| IFN a6 | JH2062 | ttcagatttccccaggaggag |
|  | JH2063 | gcaacagatgagtcctttgtgctg |
| APOBEC3F | JH2056 | cctatggtcggaacgaaagctg |
|  | JH2057 | ctgcatgacaatgggtctcagg |
| APOBEC3G | JH2054 | gctggggccaaaatttcaataa |
|  | JH2055 | ctcccactcaggtcttggctgt |
